# Malignant inguinal monophasic synovial sarcoma: report of a case and review of the literature

**DOI:** 10.1186/1477-7819-8-102

**Published:** 2010-11-21

**Authors:** Ji Xu, Jia Wang, Long Cui, Xiangru Wu

**Affiliations:** 1 Department of Surgery, Xinhua Hospital affiliated to Shanghai Jiaotong University School of Medicine, Shanghai, China 200092; 2 Department of Pathology, Xinhua Hospital affiliated to Shanghai Jiaotong University School of Medicine, Shanghai, China 200092

## Abstract

**Background:**

A synovial sarcoma (SS) is an aggressive soft tissue tumor that classically occurs in the extremities near, but rarely within large joints, in young adults. Variable symptoms and clinical manifestations may be encountered and a definite diagnosis should depend on pathological results. This poses certain difficulties in arriving at a prompt diagnosis and appropriate treatment.

**Case presentation:**

We report the case of a 68-year-old woman patient who presented an inguinal mass with swelling and pain in the right lower limb. She underwent surgery, and later received systematic intravenous chemotherapy. The pathological studies, especially the specific chromosomal translocation of a t(X;18) (p11.2;q11.2), confirmed the diagnosis as a synovial sarcoma. To the best of our knowledge, this is the first report of a monophasic synovial sarcoma in the inguinal region.

**Conclusion:**

Besides making the readership aware of the rarity of location and age of this present case, this report distinctly highlights the great value of a molecular analysis of an SYT associated genetic alteration in the diagnosis of synovial sarcoma occurring at rare sites especially when immunochemical results are equivocal.

## Background

A synovial sarcoma (SS) is an aggressive soft tissue tumor, which mainly occurs in the para-articular region of extremities with a predilection of lower limb. It usually develops in adolescents and young adults between the age of 15 and 40 years [1-3]. The detection of a reciprocal translocation between chromosomes X and 18 t(X:18) has led to the identification of an SS18 gene(also known as SYT) rearrangement being involved in the formation of a SYT-SSX fusion protein in synovial sarcomas[4-6]. With the advent of immunohistochemistry and molecular techniques, cases of synovial sarcomas have been reported in unusual location including the head and neck[7], mediastinum[8], lung[9], abdominal wall[10], intraabdominal[11], kidney[12] and retroperitoneum[13]. Isolated rare cases were mentioned in the vulva [14], skin [15], blood vessels [16] and nerves [17].

Inguinal synovial sarcomas are rare findings with only one case report in the English literature [18]. Here we report a case of an inguinal synovial sarcoma presented with pain and swelling in the right lower limb.

## Case report

**Figure 1:**
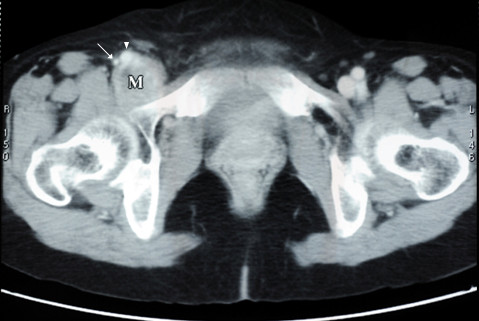
**Pre-operative CT scan showing a pelvis mass (M) adjacent to the femoral artery (arrow) and femoral vein (arrowhead)**.

**Figure 2:**
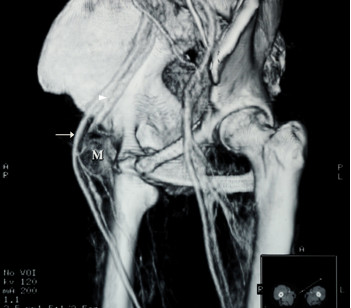
**Pre-operative CTA showing a pelvis mass (M) compressing the right femoral artery (arrow) and especially the femoral vein (arrowhead)**.

A 68-year-old woman was admitted with a complaint of swelling and pain in her right lower limb. She had felt a progressive swelling and pain for more than two months without any obvious cause. Subsequently, she was referred to a vascular surgery clinic where a mass was found in the right inguinal area by physical examination. The mass was like a goose-egg in shape, hard, fixed and couldn't disappear under pressure. A Doppler ultrasonic vessel examination suggested that there were small blood clots and reduced blood flow in the veins of the right lower limb. Correspondingly, CT and CT angiography (CTA) scans were conducted which displayed a mass in her right inguinal area with involvement of the femoral vein (Figure 1, 2). The patient did not have any family history. She had never smoked and had a normal chest X- ray exam.

**Figure 3:**
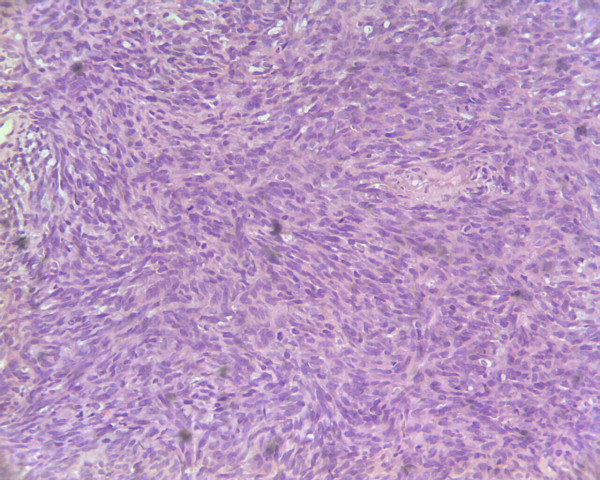
**Histological section (haematoxylin and eosin, original magnification: ×200)**. The tumor consisted of atypical spindle-shaped cells, which arranged in fascicles.

**Figure 4:**
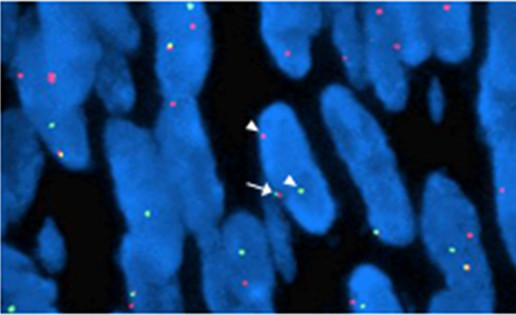
**FISH result shows a break-apart red and green signal per nucleus in SS tumor cells (arrows), indicating the presence of a t(X;18) translocation**.

A surgical excision was performed which revealed a tumor located in the right inguinal region with adhesion to the right femoral vein and artery. The tumor was completely resected with negative margin. Grossly, the specimen was 8 cm × 6 cm × 4.5 cm in size and the cut surface was firm and white-to-tan. Microscopic appearances were monomorphic, highly cellular composed of plump, spindled cells growing in short fascicles. The tumor cells had ovoid nuclei and minimal cytoplasm with frequent mitotic activity and atypical nuclei (Figure 3). Immunohistochemically, the tumor cells were positive for the epithelial membrane antigen(EMA), calretinin, cytokeratin(CK), vimentin (VIM), but negative for smooth muscle actin(SMA), muscle specific actin(MSA), CD117 and S100. The molecular analysis of the paraffin-embedded neoplasm sample by fluorescence in situ hybridization (FISH) revealed a SYT-SSX fusion transcript (Figure 4). Based on these findings, a primary malignant synovial sarcoma was diagnosed while the other most likely diagnosis of a sarcomatoid mesothelioma was excluded.

For personal reasons, the patient refused to receive any chemotherapy after the operation. Unfortunately, lung and spleen metastasis were found 15 months after operation in a routine postoperative follow-up. The patient was referred to the oncology department where she received systemic intravenous chemotherapy (ifosfamide, doxorubicin and cisplatin). However, the patient responded poorly to the treatment and finally died 9 months later due to an initially poor and subsequently rapidly worsening condition.

## Discussion

Synovial sarcomas are uncommon soft tissue tumors accounting for 5-10% of the soft tissue sarcomas. They usually develop in children and young adults and approximately 95% of SSs occur in the extremities. They can metastasize distantly, especially to the lung and lymph node [1].

Two major histologic subtypes exist: monophasic and biphasic synovial sarcomas. The classical SS has a biphasic appearance with a mixture of epithelial and spindle cells in varying proportions. The monophasic SS consisting solely of sarcomatous components is often diagnostically challenging. Synovial sarcomas typically occur in patients between the age of 15 and 40 years, rarely in the elderly. Patients commonly present with a palpable mass or swelling, associated with pain in almost half of the patients. The most common sites include the lower extremities (60%), with a special predilection for the knee area; the upper extremities (23%); the head and neck region (9%), particularly in the retropharyngeal and parapharyngeal areas; and the trunk (8.1%), most commonly involving the abdominal wall or retroperitoneum[1]. SSs occuring in the inguinal region are exceedingly rare. In a retrospective review of 20 patients, Huub[19] et al mentioned one SS case in the groin region but with no detailed documentation. The only reported case in the English literature is that of a 19-year-old Japanese female patient with the classic biphasic form of SS[18]. No immunohistochemical or further pathological analysis findings were mentioned in that brief report.

In our case, heavily stained nucleus, mitotic activity and atypical spindle-shaped cells with an increase in nucleus to cytoplasm ratio were observed under the light microscope. A panel of immunohistochemical staining was done for differential diagnosis. The spindle cells were positive for epithelial membrane antigen (EMA), calretinin, cytokeratin (CK), vimentin (VIM) and proliferating cell nuclear antigen (PCNA), but negative for smooth muscle actin(SMA), muscle specific actin(MSA), CD117 and S100. These findings helped to exclude the presence of a leiomyosarcoma, malignant peripheral nerve sheath tumor(MPNST), gastrointestinal stromal tumor(GIST). Despite of a panel of immunohistochemical staining results, however, to distinguish between sarcomatoid mesothelioma and synovial sarcoma was extremely difficult because both types of tumors share the same immunostaining results. They show an overlap in their respective immumostaining spectrum of EMA, calretinin, vimentin and S-100. To date, there exist no ideal immunohistochemical markers to reliably diagnose the SS and make an unequivocal distinction. The gold standard applied in order to confirm the diagnosis of SS is to detect the specific t(X;18)(p11.2;q11.2) translocation[4,20] at molecular level. The t(X;18)(p11.2;q11.2) translocation is a cytogenetic hallmark of the SS and is present in more than 90% of the cases[21]. The SS18-SSX fusions do not seem to occur in other types of sarcomas. To determine whether the SYT-SSX fusion transcript associated with t(X;18) translocation is present, a dual-color break-apart fluorescence in situ hybridization (FISH) assay for SYT gene disruption was performed in paraffin-embedded tumor material, which showed a positive result. As the t(X;18) translocation is unique to the SS, its identification provided the definite diagnosis for this case. We consider this FISH assay described by Surace et al [22], a preferred clinical method in the diagnosis, especially when sufficient quality of RNA for PCR could not be extracted. Apart from its diagnostic value, this translocation fusion type was found to be the single most significant prognostic factor by multivariate analysis in SS patients [23]. However, a European retrospective analysis found that the most important factor in determining the prognosis of these patients is histological grade, and not SYT-SSX fusion type [24].

Surgery is the mainstay treatment. Chemotherapy and radiation therapy should be considered as additional treatment options or may be utilized in cases of relapse. Prognosis is poor with local recurrence and metastases in almost 50% of the patient [1]. Metastases occur mainly to lungs as was the case for this patient. Since Ifosfamide based chemotherapy has been shown to produce notable responses in the treatment of metastatic SS [25], however, its effect on this patient was not satisfactory. Finally, despite the surgery and chemotherapy she had received, our patient has only survived for less than two and a half years after her initial clinical presentation.

Currently, with known genetic alterations in SSs, new therapies targeting DNA or protein are under investigation. Vaccine of SYT-SSX junction peptide was tested in a pilot study [26]. Recent studies shows retinoic acid and its derivatives could induce the differentiation of SS cell lines and inhibit cell growth both in vitro and in vivo [27]. However, the therapeutic efficacy is still under evaluation.

In conclusion, this is the first report of a monophasic synovial sarcoma in the inguinal region. We report this unusual case because of the tumor location and patient's old age. The case illustrates possible differential diagnoses before the treatment. It also highlights the value of molecular analysis in ascertaining the diagnosis of a synovial sarcoma at a rare site, especially when immunohistochemical results are equivocal.

## Consent

Written informed consent was obtained from the patient for publication of this case report and accompanying images. A copy of the written consent is available for review by the Editor-in-Chief of this journal.

## Competing interests

The authors declare that they have no competing interests.

## Authors' contributions

JX performed surgery on the patient and participated in designing the report and also was involved in data acquisition and interpretation, writing initial drafts and subsequent revisions and undertook review of literature on topic. JW made a substantial contribution regarding conception of report, made many suggestions regarding pathological issues contained in the report and undertook review of pertinent literature on topic. XW made a substantial contribution regarding pathological molecular analysis. LC made a substantially contributed to the concept of this case report, supervised the work and drafts of the case report prepared by the first and second authors. All authors read and approved the final version of the article to be submitted for publication.
